# Case Report: Primary cardiac diffuse large B-cell lymphoma with sick sinus syndrome and literature review on disease management and therapeutic strategies

**DOI:** 10.3389/fonc.2025.1538786

**Published:** 2025-07-04

**Authors:** Xiaofen Qiu, Li Zhang, Shaojun Zhou, Wenbin Qian, Yun Liang, Huiqing Qiu, Xianggui Yuan

**Affiliations:** ^1^ Department of Hematology and Medical Oncology, The Second People’s Hospital of Quzhou, Quzhou, China; ^2^ Department of Hematology, The Second Affiliated Hospital, Zhejiang University School of Medicine, Hangzhou, China; ^3^ National Clinical Research Center for Hematologic Diseases, The First Affiliated Hospital of Soochow University, Suzhou, China

**Keywords:** primary cardiac lymphoma, diffuse large B-cell lymphoma, extranodal lymphoma, sick sinus syndrome, orelabrutinib, polatuzumab vedotin

## Abstract

**Background:**

Primary cardiac diffuse large B-cell lymphoma (DLBCL) is a rare but clinically challenging extranodal lymphoma. Diagnosis and management are often complicated due to its nonspecific symptoms and rarity.

**Case Report:**

We reported a case of a 73-year-old male who initially presented with chest pain, high fever, dizziness, and amaurosis. Preliminary diagnostic assessments suggested sick sinus syndrome, necessitating the implantation of a dual-chamber pacemaker, and revealed a large mass in the interatrial septum. An endomyocardial biopsy confirmed the diagnosis of primary cardiac DLBCL. Initial treatment with R-miniCHOP chemotherapy yielded a partial response. However, due to treatment-related complications (grade 4 neutropenia and pneumonia), a change in the therapeutic regimen to OR-GemOx chemotherapy was made, leading to complete remission. A year later, the patient experienced a relapse, requiring a salvage treatment of the Pola-BR chemotherapy regimen, which again resulted in complete remission. Additionally, this review examines an in-depth literature review on the management and therapeutic strategies for this entity, focusing on the treatment recommendations for relapse/refractory disease.

**Conclusion:**

Prompt diagnosis and effective management are crucial in treating primary cardiac DLBCL. While the emergence of new drugs has improved the prognosis by offering higher efficacy and fewer side effects, clinicians should be vigilant about potential cardiotoxicities.

## 1 Introduction

Cardiac lymphoma refers to lymphoma affecting the myocardium or pericardium, and it is a rare form of extranodal lymphoma. It is categorized into primary cardiac lymphoma (PCL) and secondary cardiac lymphoma (SCL) based on cellular origin. PCL is exceptionally rare, accounting for approximately 1.3% of all primary cardiac tumors in autopsies and 0.5% of extranodal lymphomas (1). In contrast, secondary cardiac lymphoma (SCL) is more prevalent, constituting about 20% of secondary cardiac tumors. PCLs are remarkably invasive lymphomas. The predominant histopathological type is diffuse large B-cell lymphoma, which accounts for 80% of all cases. The prognosis of PCLs is generally poor, with the median survival time of just 2.2 years according to a U.S. countrywide cancer database (2). Due to its non-specific symptoms, early diagnosis of PCLs is often challenging. Given its aggressive nature, rapid progression, and poor prognosis, timely and effective diagnosis, management, and treatment are of paramount importance.

In this report, we presented the case of a 73-year-old male diagnosed with primary cardiac DLBCL. He initially exhibited symptoms such as chest pain, continuous high fever, and progressive amaurosis. We provide a detailed account of the disease course, the diagnostic procedures employed, and the therapeutic management, which included various chemotherapy regimens such as R-miniCHOP (rituximab, cyclophosphamide, liposomal doxorubicin, vincristine, dexamethasone), OR-GemOx (orelabrutinib, rituximab, gemcitabine, oxaliplatin), and Pola-BR (polatuzumab vedotin, bendamustine, rituximab). Furthermore, we conduct an in-depth review of the existing literature to offer valuable clinical insights for the better understanding and management of this entity, focusing on the treatment recommendations for relapse/refractory disease.

## 2 Case report

**Figure 1:**
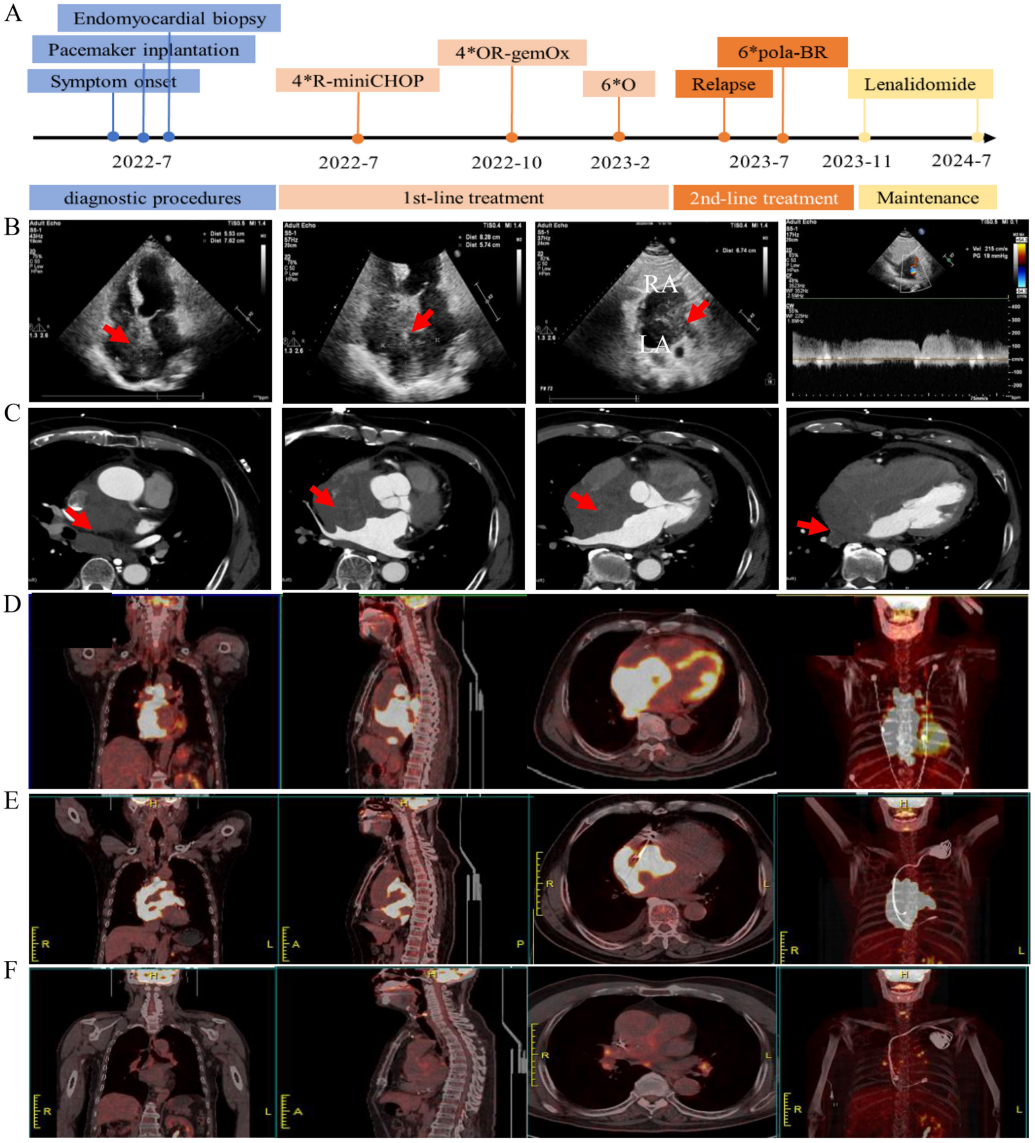
Radiological imaging before and after the treatment. **(A)** Timeline of clinical events, and interventions. **(B)** Transthoracic echocardiogram showing a large mass (red arrow) infiltrating the interatrial septum. Panel 1–3 show the apical 4-chamber and subxiphoid two-chamber views of the interatrial septal mass, panel 4 shows pulse-wave doppler imaging of the mass at the junction of the superior vena cava and the right atrium with increased blood flow velocity. **(C)** Contrast-enhanced CT scan shows a large mass (red arrow) in the middle mediastinum invading the left and right atria. **(D)** PET/CT shows increased 18-FDG-uptake heart masses invading surrounding structures at diagnosis. **(E)** PET/CT shows increased 18-FDG-uptake heart masses at relapse. **(F)** PET/CT shows no evidence of lymphoma after treatment for disease relapse.

In July 2022, a previously healthy 73-year-old male was admitted to our cardiology department presenting with chest pain for over ten days. The pain began after exertion, lasting for several seconds, and showed minimal improvement with anti-anginal therapy. Three days before hospital admission, the patient started experiencing a repeated high fever, progressive dizziness and amaurosis fugax. An electrocardiogram (ECG) revealed sinus or ectopic atrial rhythm, multiple atrial premature beats, non-specific intraventricular conduction delay, low-voltage limb leads, and mild ST-segment elevation in inferior wall leads. A 24-hour Holter monitoring indicated multiple atrial premature beats and frequent sinus arrest >4s, suggestive of sick sinus syndrome. An echocardiography showed a heterogeneous hypoechoic mass in the interatrial septum, measuring approximately 5.74*6.74*8.28cm (**Figure 1B**). The left ventricular ejection fraction (LVEF) was 48%. A contrast-enhanced cardiac computed tomography (CT) demonstrated mild enhanced multiple masses in the middle mediastinum invading the left and right atria, the superior vena cava, aortic root, and right pulmonary vein (**Figure 1C**). Laboratory examination revealed NT-ProBNP 3420 (range, <125) pg/mL, hemoglobin 109 g/L (range, 120-160), and lactate dehydrogenase (LDH) 245 U/L (range, 176-235) U/L. Cardiac biomarkers (troponin I and CK-MB) were all normal. Blood cultures were repeatedly negative during the febrile episodes. Inflammatory markers (C-reactive protein and procalcitonin) were within normal limits. Upon admission, the patient received intensive antibiotic therapy, but his fever persisted, and symptoms of dizziness and amaurosis did not alleviate. Consequently, a dual-chamber pacemaker was implanted. After the operation, the patient developed persistent atrial fibrillation refractory to amiodarone therapy, and low-molecular-weight heparin was initiated to prevent the thrombosis.

**Figure 2:**
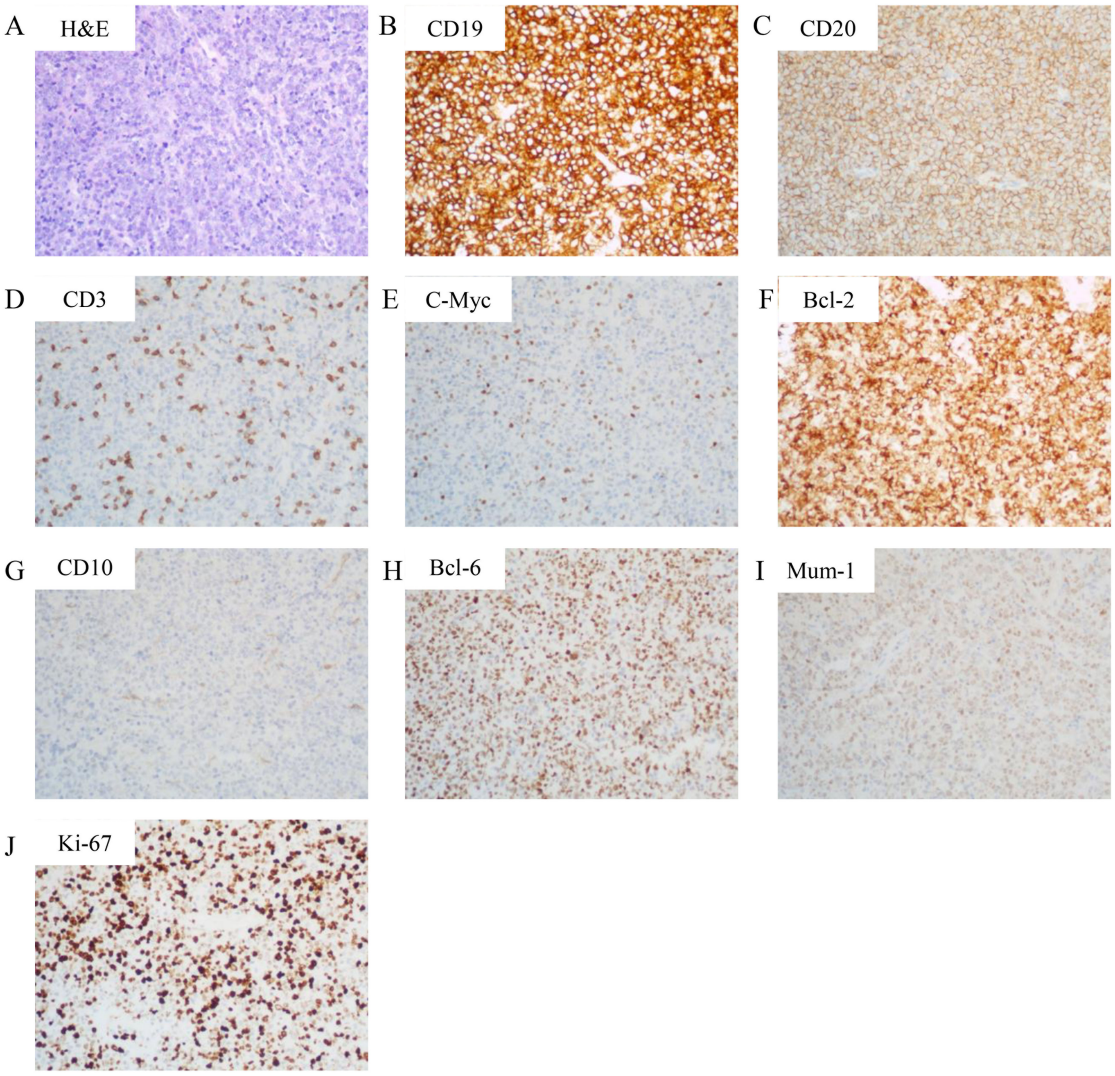
Histopathological and immunohistochemical features. **(A)** Hematoxylin and eosin (H&E) staining (200× magnification) demonstrating sheets of large atypical lymphoid cells with vesicular chromatin and prominent nucleoli, consistent with diffuse large B-cell lymphoma. **(B-I)** Immunohistochemical staining (200× magnification) revealing: **(B)** Strong CD19 positivity in neoplastic cells **(C)** Diffuse membranous CD20 expression; **(D)** CD3 negativity (internal control: background T-lymphocytes show positive staining); **(E)** c-Myc expression in 10% of tumor cells; **(F)** Bcl-2 overexpression in 90% of neoplastic cells; **(G)** CD10 negativity; **(H)** Nuclear Bcl-6 positivity; **(I)** MUM1 nuclear expression. **(J)** Ki-67 staining showing high proliferative index (70%+).

To determine the nature of the cardiac mass, an angiography-guided endomyocardial biopsy was performed. The pathological examination revealed diffuse lymphocytic proliferation. Immunohistochemistry results were as follows: ALK/P80 -, c-Myc 10%+, Bcl-6 +, CD10 -, CD19 +, CD20 +, CD21 -, CD23 -, CD3 -, CD30 -, CD35 -, CD45(LCA) +, CD45RO -, CD5 -, CD79a +, Cyclin D1 -, Ki-67 70%+, Mum-1 +, SOX11 -, Bcl-2 90%+, leading to a diagnosis of DLBCL, non-GCB subtype (**Figure 2**). Following this diagnosis, the patient was transferred to the hematology department for further treatment. A PET-CT scan revealed heart masses invading and compressing surrounding structures with abnormal ^18^F-FDG uptake (SUVmax=44.2), and slightly enlarged hilar lymph nodes with increased ^18^F-FDG uptake (**Figure 1D**). Bone marrow biopsy showed no lymphomatous involvement. A final diagnosis of primary cardiac DLBCL was made.

Upon diagnosis confirmation, the patient received intravenous dexamethasone 10mg QD and hydration/alkalization to prevent tumor lysis syndrome, followed by R-miniCHOP chemotherapy (Rituximab 375 mg/m² (d0), cyclophosphamide 600 mg/m² (d1), liposomal doxorubicin 40 mg/m² (d1), vincristine 1 mg (d1), prednisone 60 mg (d1-5)), due to the patient’s advanced age and concern for cardiotoxicity from full-dose anthracyclines (3, 4). After one cycle of chemotherapy, ventricular pacing burden decreased, and atrial fibrillation resolved. The patient reported significant improvement in chest pain and dizziness. A partial response based on PET/CT was observed after four cycles of chemotherapy, as the cardiac tumor size reduced from approximately 5.74*6.74*8.28cm to 3.76*6.29*3.19cm as well as ^18^F-FDG uptake reduced. However, treatment tolerance was suboptimal. Despite the preventive use of pegylated granulocyte colony stimulating factor, the patient developed neutropenia pneumonia requiring hospitalization after the third and fourth cycle of chemotherapy. Thereafter, the chemotherapy regimen was changed to OR-GemOx (Orelabrutinib 150 mg QD, rituximab 375 mg/m² (d0), gemcitabine 1000 mg/m² (d1), oxaliplatin 100 mg/m² (d1)) for 4 cycles, leveraging orelabrutinib’s cardiac safety profile (5), and metabolic complete remission was achieved. The patient reported that fatigue during R-miniCHOP impacted daily activities, but OR-GemOx was better tolerated. Patient-reported grade 2 fatigue (CTCAE v5.0) resolved during OR-GemOx treatment. Then, maintenance therapy with orelabrutinib was initiated for six months.

In July 2023, the patient was admitted with a one-week history of chest tightness. A chest enhanced CT scan revealed a tumor around the superior vena cava and the right middle lobe bronchus. A PET-CT scan showed hypermetabolic masses confined to the heart (SUVmax=34.25), indicating disease recurrence (**Figure 1E**). The patient’s condition precluded a biopsy again. Consequently, four cycles of the Pola-BR regimen [Polatuzumab vedotin 1.8 mg/kg (d1), bendamustine 70 mg/m² (d1-2), rituximab 375 mg/m² (d1)] were administered (6), achieving metabolic complete remission (**Figure 1F**). After two consolidation cycles of Pola-BR regimen, the patient was maintained with lenalidomide 10 mg/day. The patient underwent a structured cardiovascular follow-up protocol. Echocardiography, ECG, and NT-proBNP every 3 months, cardiac MRI (post-pacemaker adjustment to MRI-safe mode) to assess myocardial infiltration and fibrosis every 6 months, and symptom-driven troponin testing and Holter monitoring for arrhythmia recurrence. At the last follow-up in July 2024, the patient remained in a complete remission. His LVEF improved to 65%, with normalization of NT-proBNP and troponin-I levels post-Pola-BR. During the treatment, no cardiac events were observed. Timeline of clinical events and interventions were shown in **Figure 1A**.

## 3 Discussion

Primary cardiac DLBCL is a rare and aggressive form of extranodal lymphoma often characterized by a poor prognosis. This case presents considerable diagnostic and therapeutic challenges encountered when dealing with this entity. The patient’s initial nonspecific symptoms caused a delay in diagnosis. Furthermore, biopsy was delayed by symptomatic sick sinus syndrome, necessitating pacemaker implantation prior to invasive procedures. Initial imaging suggested angiosarcoma or metastatic carcinoma. DLBCL was confirmed via biopsy after excluding infectious etiologies (e.g., infective endocarditis via repeated blood cultures) and non-lymphomatous tumors (e.g., cardiac myxoma with pathological features). The first line of treatment R-miniCHOP was chosen over full-dose R-CHOP due to age-related cardiotoxicity concerns. However, although this regimen demonstrated efficacy, but it was complicated by severe chemotherapy-related complications (grade 4 neutropenia and pneumonia). Then OR-GemOx was substituted after R-miniCHOP-induced neutropenia pneumonia, leveraging orelabrutinib’s cardiac safety profile, which was successful in achieving complete remission. However, a disease recurrence called for another change in the chemotherapy regimen to Pola-BR, which also resulted in complete remission. To prevent further relapses, lenalidomide was initiated as maintenance therapy. Novel agents with improved safety profiles and higher efficacy have significantly improved the patient’s prognosis.

We collected primary cardiac DLBCL case reports published from January 2009 to January 2024, collected statistics from each patient, and performed a systematic analysis (**Table 1**). According to the narrow definition given by the WHO in 2022 as “extranodal lymphoma involving only the heart or the pericardium”, 81 cases of primary cardiac DLBCL cases in 76 reports were included (**Appendix Table S1**). Signs and symptoms can vary based on factors such as tumor location, size, growth rate, degree of invasion, and friability. Most symptoms are nonspecific. Dyspnea (64%), chest pain (19%) and palpitation (19%) are the most common symptoms reported. Congestive heart failure characterizes 16% of clinical presentation. Other less common manifestations include cardiogenic shock (5%), and tumor embolization (2%) and cardiac tamponade (2%). Various types of arrhythmias are also not uncommon. They often manifest as atrial fibrillation (15%), and varying degrees of atrioventricular block (21%), while sick sinus syndrome is relatively rare. One case report described a patient with cardiac lymphoma that caused atrial fibrillation and sick sinus syndrome, necessitating pacemaker therapy. After chemotherapy, ventricular pacing burden was reduced without atrial fibrillation (7). Another case report presented a patient with cardiac lymphoma and sick sinus syndrome who were successfully treated via chemotherapy without pacemaker implantation (8). This case report outlined a patient with primary cardiac DLBCL and sick sinus syndrome that did not respond to conventional treatment. However, post-chemotherapy and tumor control, the arrhythmias resolved. Although pacemaker implantation may not be entirely avoidable in case where sick sinus syndrome is present, appropriate treatment of the cardiac lymphoma may potentially restore conduction partially or completely, suggesting that the sick sinus syndrome was likely due to the reversible invasion of the sinus node by the lymphoma.

**Table 1:** Summary of characteristics and outcomes of primary cardiac DLBCL cases reported from 2009 to 2024.

Characteristics	Patient number (%) (*n* = 81)
Age, years, median (range)	65 (29-89)
Sex	
Male	49 (60%)
Female	32 (40%)
Symptom/findings	
Dyspnea (%)	52 (64%)
Chest pain (%)	15 (19%)
Heart failure	13 (16%)
Palpitation (%)	13 (16%)
B symptoms	11 (14%)
Syncope (%)	9 (11%)
Fatigue (%)	9 (11%)
Cough (%)	8 (10%)
Chest distress	8 (10%)
SVC syndrome (%)	7 (9%)
Edema (%)	6 (7%)
Shock (%)	4 (5%)
Dizziness (%)	3 (4%)
Pulmonary embolism (%)	2 (2%)
Cardiac tamponade (%)	2 (2%)
Arrhythmias	
Complete AV block (%)	12 (15%)
Atrial fibrillation (%)	12 (15%)
Ventricular tachycardia (%)	4 (5%)
Second degree AV block (%)	3 (4%)
First degree AV block (%)	2 (2%)
Ventricular fibrillation (%)	1 (1%)
Supraventricular tachycardia (%)	1 (1%)
Mass location	
Right atrium (%)	60 (74%)
Right ventricle (%)	44 (54%)
Pericardial effusion (%)	26 (32%)
Left atrium (%)	9 (11%)
Interatrial septum (%)	8 (10%)
Left ventricle (%)	8 (10%)
Interventricular septum (%)	3 (4%)
Others (%)	12 (15%)
Methods of diagnosis	
Open chest biopsy (%)	31 (38%)
Endomyocardial biopsy (%)	24 (30%)
CT-guided biopsy (%)	7 (9%)
Percutaneous TTE-guided biopsy (%)	6 (7%)
Pericardial fluid (%)	3 (4%)
Autopsy (%)	2 (2%)
Transesophageal echocardiography (%)	2 (2%)
Mediastinoscopy biopsy (%)	1 (1%)
Unspecified (%)	5 (6%)
Cell of Origin Subtype	
GCB (%)	5 (6%)
Non-GCB (%)	11 (14%)
Unspecified (%)	65 (80%)
Cardiac complications	
CNS Relapse	6 (7%)
Arrhythmias	1 (1%)
Sudden cardiac death	1 (1%)
Median PFS (range)	36 (13.5-58.5) months
2y-PFS rate	68.6%
Median OS (range)	Not reached
3y-OS rate	75.8%

PFS, Progression free survival; OS, Overall survival; GCB, Germinal center B-cell-like lymphoma; non-GCB, non-germinal center B-cell-like lymphoma; TTE, Transthoracic echocar-diography.

In case of suspected cardiac involvement, trans-thoracic echocardiography is the first-line imaging modality for screening. Computed tomography (CT) provides a more detailed view, capturing the morphology, location, and extent of cardiac neoplasms. Magnetic resonance imaging (MRI), on the other hand, offers superior images identifying the anatomy, blood flow, and cardiac function. Both CT and MRI offer superior contrast resolution compared to echocardiography, providing diagnostic and differential diagnoses of PCLs from angiosarcoma, metastatic tumors, and myocarditis. The gold standard to stage the disease, though, is PET/CT, which assesses the overall tumor burden and extent, detects latent cardiac involvement and determines the primary nature of cardiac lesions. It was reported that PET/CT parameters and cutoff values (SUVmean>5.17) together can help to distinguish PCLs from primary cardiac angiosarcomas with high sensitivity and specificity (9). However, there are considerable overlaps in imaging characteristics among cardiac tumors, making the initial diagnosis based on imaging features challenging. Hence, these different imaging modalities should be seen as complementary tools rather than competitive alternatives.

The definitive diagnosis of PCLs relies on pathological biopsy. Traditional open-chest biopsy (38%) carries significant risks, leading to a shift towards less invasive procedures in recent years. These include transvenous angiography-guided endomyocardial biopsy (30%), trans-thoracic CT-guided biopsy (9%), trans-thoracic echocardiography-guided biopsy (7%), transesophageal echocardiography-guided biopsy (2%), and mediastinoscopy (1%). In cases where pericardial effusion is present, cytological examination can also aid in diagnosis. The transvenous angiography-guided endomyocardial biopsy is particularly suitable for masses in the chambers or in the diffusely thickened myocardium. Recent reports from the European Society of Cardiology have shown that endomyocardial biopsy, predominantly right ventricular, carries a low complication rate and no procedure-related fatalities (10).

Currently, there are no guidelines available regarding treatment for primary cardiac DLBCL, necessitating a multidisciplinary approach involving cardiologists, oncologists, and hematologists. Generally, cardiac DLBCL is sensitive to chemotherapy. The first line treatment usually involves a combination of drugs, most commonly R-CHOP (Rituximab, cyclophosphamide, doxorubicin, vincristine, and prednisone). However, our retrospective analysis revealed 8.6% of treatment-related severe cardiac complications in PCL patients treated with R-CHOP-like regimens, suggesting that rigorous monitoring protocols coupled with risk (**Appendix Table S1**). Therefore, individualized treatment plans based on patient response and tolerance are highly recommended, with a focus on avoiding cardiotoxic drugs and being vigilant to cardiac events. Cardiac DLBCL is also sensitive to radiation therapy, but due to potential cardiac side effects, radiation is generally limited to cardiac masses that do not respond to chemotherapy. Cardiac DLBCL is highly likely to recurrence and eventually become refractory. Regular follow-up is crucial for monitoring signs of recurrence or progression.

Once the disease relapses, the prognosis is extremely poor. However, limited treatment recommendations are available so far for relapse/refractory(R/R) cardiac DLBCL. Bruton’s tyrosine kinase (BTK) inhibitors, either alone or in combination, have been identified as a promising therapeutic option for systemic DLBCL (11, 12), particularly for cases with extranodal lesions and MCD genetic subtype. However, common cardiac side effects such atrial fibrillation or flutter (16%), cardiac failure (5%) and symptomatic ventricular arrhythmias (3%), may limit their use in R/R cardiac DLBCL. Second-generation BTK inhibitors such as orelabrutinib may minimize these cardiac events. Recent studies have shown that orelabrutinib combined with chemotherapy or immunotherapy is effective for R/R systemic DLBCL with fewer cardiac events (15.8%) (5). Polatuzumab vedotin, an antibody drug conjugates, combined with bendamustine and rituximab (Pola-BR) has yielded significantly higher CR rates and survival in R/R systemic DLBCL (6). However, a pharmacovigilance study based on the Food and Drug Administration Adverse Event Reporting System (FAERS) database indicated that it has been associated with cardiotoxicity such as cardiac failure, supraventricular tachyarrhythmias, and cardiomyopathy (13). Bispecific antibodies (Glofitamab, Blinatumomab) have shown notable success in R/R systemic DLBCL (14), but they can lead to mild sinus tachycardia (5-6%), and rare but severe cardiac adverse events (<0.5%), including myocardial infarction, atrial arrhythmias, congestive heart failure, cardiac arrest, and pericardial effusion (15). The FDA has also approved three CAR-T cell therapies for R/R systemic DLBCL. However, CAR-T cell therapy can also induce cardiac complications, such as cardiomyopathy (10.8%), heart failure (15%), arrhythmias (12.2%), myocardial infarction (7.1%), shock (50%), cardiac arrest (2.2%), and cardiovascular death (up to 4.3%) (16). A rare case with secondary cardiac DLBCL receiving CAR-T treatment achieved transient metabolic complete response but suffered from severe fulminant cardiotoxicity (17). In the era of novel agents, while they may effectively improve the treatment outcome for cardiac DLBCL, it is crucial to be mindful of their cardiac toxicity, warranting vigilant monitoring and tailored management. This is the first reported case utilizing orelabrutinib- and polatuzumab-based regimens for cardiac DLBCL, demonstrating feasibility in elderly patients. However, lack of molecular profiling (e.g., *MYD88* and *CD79B* mutation) and short follow-up (24 months) preclude long-term efficacy conclusions.

In conclusion, primary cardiac DLBCL is a rare and challenging malignancy that requires prompt diagnosis and aggressive multimodality treatment. Close monitoring and individualized treatment plans are critical to optimize outcomes and prevent relapses.

## Data Availability

The original contributions presented in the study are included in the article/**Supplementary Material**. Further inquiries can be directed to the corresponding authors.
