# Supplementary material for: Case Report: Primary cardiac diffuse large B-cell lymphoma with sick sinus syndrome and literature review on disease management and therapeutic strategies
Source: Front Oncol. 2025 Jul 4;15:1538786. doi: 10.3389/fonc.2025.1538786 (PMC12270880; doi:10.3389/fonc.2025.1538786)
Supplement: Supplementary file 1 [file DataSheet1.pdf]

Appendix Table S1. Details of case reports of primary cardiac diffuse large B cell lymphoma since 2009 to 2024

Reference, year	No. patients	Age	Sex	Pathological type	Localization in heart	Symptoms	Arrhythmias	Pathological Diagnostic tool	Therapy	First-line Chemotherapy (Response)	Salvage Chemotherapy	Cardiac complications	Outcome
Su et al. (2009)[1]	1	43	M	NA	Right atrium, interatrial septum, left atrium	Syncope	Complete AV block	Endometrial biopsy	NA	NA	NA	NA	NA
Dellas et al. (2009)[2]	1	56	M	NA	Right ventricle	Shock	Ventricular fibrillation	Angiography biopsy	Chemotherapy	R-CHOP	NA	NA	PFS>14m
Utsunomiya et al. (2009)[3]	1	76	F	NA	Right atrial septum	Palpitation and dyspnea	NA	Open chest biopsy	Chemotherapy	R-CHOP	NA	NA	PFS>24m
Sankaranarayanan et al. (2009)[4]	1	84	M	NA	Right atrium, right ventricle	Chest tightness, dyspnea	Non-ST-elevation myocardial infarction, atrial fibrillation	Autopsy	NA	NA	NA	NA	OS=8d
Schell et al. (2009)[5]	1	60	M	GCB	Right atrium	Superior vena cava syndrome	NA	Open chest biopsy	Chemotherapy	R-CHOP	NA	NA	PFS>14m
Patel et al. (2010)[6]	1	50	M	GCB	Aortic valve replacement	Aortic valve replacement vegetations	NA	Coronary arteriogram biopsy	Chemotherapy	R-CHOP(CR)	NA	Rupture of the bioprosthesis	PFS=6m OS=6m
Mariani et al. (2010)[7]	1	76	F	NA	Right atrium, right ventricle	Syncope and dyspnea	NA	Subxiphisternal biopsy	Chemotherapy	R-CHOP	NA	NA	PFS>60m
Park et al. (2010)[8]	1	52	F	NA	Right atrium	Dyspnea, fatigue	NA	Echocardiography-	Chemotherapy	R-CHOP	NA	NA	NA

Reference, year	No. patients	Age	Sex	Pathological type	Localization in heart	Symptoms	Arrhythmias	Pathological Diagnostic tool	Therapy	First-line Chemotherapy (Response)	Salvage Chemotherapy	Cardiac complications	Outcome
								guided biopsy					
Cho et al. (2010)[9]	1	71	F	NA	Right atrium	Dyspnea, swelling	NA	Open chest biopsy	Chemotherapy	R-CHOP	NA	NA	PFS>4m
Cho et al. (2010)[10]	1	82	F	NA	Pleural effusion.	Dizziness, palpitation, and dyspnea.	Atrial fibrillation Sick Sinus Syndrome	Open chest biopsy	Chemotherapy	R-CHOP	NA	NA	PFS>6m
Cho et al. (2010)[11]	1	55	M	NA	Right atrium, periaortic space, and left atrium	Dyspnea, chest discomfort, B symptoms (night sweats)	Second degree AV block	Open chest biopsy	Chemotherapy	R-CHOP	NA	NA	PFS>6m
Lee et al. (2010)[12]	1	51	M	NA	Right atrium, right ventricle	Syncope, Dyspnea	Polymorphic ventricular tachycardia	Percutaneous TTE-guided biopsy	Chemotherapy	Anthracycline-based polychemotherapy	NA	NA	PFS>7m
Chen et al. (2010)[13]	1	54	M	NA	Right atrium,	Abdominal distension, edema of the lower extremities, distress	NA	Open chest biopsy	Chemotherapy	R-CHOP	NA	NA	PFS>36m
Nishizawa et al. (2010)[14]	1	68	F	NA	Right atrium	Heart failure	NA	Open chest biopsy	Chemotherapy	R-CHOP	NA	NA	PFS=3m

Reference, year	No. patients	Age	Sex	Pathologic al type	Localizati on in heart	Symptoms	Arrhythmia s	Pathologic al Diagnostic tool	Therapy	First-line Chemothera py (Response)	Salvage Chemothera py	Cardiac complication s	Outcome
Lin et al. (2010)[15]	1	42	M	NA	Left atrial septum and interatrial septum	Syncope	Complete atrioventricul ar block	Endomyoc ardial biopsy	Chemother apy	R-CHOP	NA	Sudden cardiac death	PFS=4m OS=4m
Minamimo to et al. (2011)[16]	1	66	F	NA	Left ventricle	Fever and dry cough	NA	Open chest biopsy	Chemother apy+Radiat ion	R-CHOP(CR)	NA	NA	PFS>52m
Bambury et al. (2011)[17]	1	70	F	NA	Pericardial effusion, right ventricle	Dyspnea	NA	Mediastino scopy biopsy	Chemother apy	R-CHOP	NA	VT	PFS>7m
Matsushita et al (2012)[18]	1	67	M	NA	Right atrium, pericardial effusion	Dyspnea, fatigue, leg edema	NA	Open chest biopsy	Resection + Chemother apy	R-CHOP	NA	NA	NA
Yoshihara et al (2013)[19]	1	86	F		Right atrium, Pericardial effusion	Heart failure	NA	Autopsy	NA	NA	NA	NA	OS<1m
Toshiharu et al. (2013)[20]	1	56	M	NA	Right atrium, right ventricle pericardial effusion	Palpitation, dyspnea, cardiogenic shock	NA	Transesoph ageal echocardio graphy	Chemother apy	NA	NA	NA	PFS>36m
Kawammu ra et al. (2013)[21]	1	60	M	NA	Right ventricle, Right atrium, tricuspid valve	Dyspnea	None	Open chest biopsy	Chemother apy	R-CHOP+ASC T(PR)	NA	NA	PFS>12m
Shah et al. (2014)[22]	1	62	M	NA	Interatrial septum,	B symptoms	NA	Endomyoc ardial	Chemother apy	R-CHOP（CR）	NA	NA	PFS>12m

Reference, year	No. patients	Age	Sex	Pathological type	Localization in heart	Symptoms	Arrhythmias	Pathological Diagnostic tool	Therapy	First-line Chemotherapy (Response)	Salvage Chemotherapy	Cardiac complications	Outcome
					tricuspid valve	(Weight loss, Fatigue, Night sweats), Heart failure.		biopsy					
Kim et al. (2014)[23]	1	43	M	NA	Pericardial effusion, thickening of right and left ventricular	Dyspnea, Heart failure	NA	Endomyocardial biopsy(negative), CT-guided needle biopsy	Chemotherapy	NA	NA	NA	PFS> 6m
Jung et al. (2014)[24]	1	62	M	NA	Right atrium	Dyspnea, syncope	NA	Open chest biopsy	Chemotherapy	R-CHOP(CP)	NA	CNS Relapse	PFS=5m
Montoro et al. (2014) [25]	1	54	F	NA	NA	Dyspnea., Heart failure	None	Endomyocardial biopsy	Chemotherapy	R-VACOP-B)	NA	CNS relapse	PFS=4m OS=7m
	1	59	F	NA	Right atrium, right ventricle	Dyspnea, chest pain.	NA	Endomyocardial biopsy	Chemotherapy	R-CHOP+MTX (CR)	NA	NA	PFS=5m; OS>8m
Nijjar et al. (2014)[26]	1	55	M	NA	Right atrium, right ventricle	Dyspnea, edema	NA	Trans-thoracic echocardiography	Chemotherapy	R-CHOP	NA	NA	PFS>34m
Jonavicius et al. (2015) [27]	1	48	F	NA	Left and right atrium, Left ventricle through interventricular septum	Heart failure Dyspnea Cyanosis B-symptoms	NA	Open chest biopsy	Chemotherapy	NA	NA	NA	NA
	1	64	F	Non-GCB	Right	Heart	NA	Open chest	Palliative	NA	NA	NA	Died soon

Reference, year	No. patients	Age	Sex	Pathological type	Localization in heart	Symptoms	Arrhythmias	Pathological Diagnostic tool	Therapy	First-line Chemotherapy (Response)	Salvage Chemotherapy	Cardiac complications	Outcome
					atrium, right ventricle, Pericardial effusion	failure, Chest pain		biopsy	surgery				after surgery
Riccoli et al. (2015) [28]	1	62	M	NA	Right atrium	SVC syndrome	NA	Endomyocardial biopsy	Chemotherapy	R-CHOP(CR)	NA	NA	PFS>8m
Jandali et al. (2015) [29]	1	62	M	NA	Pericardium, right atrium, tricuspid valve, interatrial septum, right ventricle	Fatigue, Dyspnea, B symptoms (weight loss, night sweat)	NA	Endomyocardial biopsy	Chemotherapy	R-CHOP	NA	NA	NA
Hong et al. (2015)[30]	1	58	M	NA	Right ventricle, right atrium	Asymptotic	NA	Open chest biopsy	Chemotherapy	R-CHOP(CR)	NA	NA	PFS>4m
Page et al (2016)[31]	1	54	,M	NA	Left ventricle and left atrium wall thickening	Chest pain, syncope, heart failure	NA	Endomyocardial biopsy	Chemotherapy	R-CHOP(CR)	NA	Heart failure, pulmonary embolism, ventricular tachycardia, ventricular d fibrillation	PFS>10m
Moss et al (2016) [32]	1	67	M	NA	Left ventricular	None	NA	Endoscopic approach	Chemotherapy	R-CHOP	NA	NA	PFS>5m
Liu et al (2016) [33]	1	61	F	NA	Right atrium, right ventricular, pericardial effusion,	Palpitation, dyspnea	None	Open chest biopsy	None	NA	NA	Heart failure	OS=2w

Reference, year	No. patients	Age	Sex	Pathological type	Localization in heart	Symptoms	Arrhythmias	Pathological Diagnostic tool	Therapy	First-line Chemotherapy (Response)	Salvage Chemotherapy	Cardiac complications	Outcome
					tricuspid annulus								
	1	74	M	NA	Right atrium, interatrial septum, tricuspid annulus	Dyspnea	Atrial fibrillation	Trans jugular intracardiac mass biopsy	None	NA	NA	Cardiac arrest	OS=1w
Ciancarella et al. (2016)[34]	1	76	F	NA	Right atrium right ventricular, pericardial effusion	Dyspnea, chest pain	NA	Open chest biopsy	Chemotherapy	R-CHOP	NA	NA	NA
Singh et al. (2016) [35]	1	63	F	NA	Right atrium and right ventricle	Chest pain, fatigue, and dyspnea, pulmonary embolism	Second-degree atrioventricular (AV) block	Open chest biopsy	Chemotherapy, prophylactic automated implantable cardioverter-defibrillator (AICD)	R-CHOP(CR)	R-CHOP(CR)	NA	PFS=84m, OS=87m
Cereda et al. (2017) [36]	3	46,74,70	2M, 1F	NA	Right side of heart (3), pericardial effusion (1)	Dyspnea(3),chest pain（3）, palpitations(3), cough(3),Fever（2）	NA	NA	Chemotherapy	R-CHOP(CR)	NA	Ventricular tachycardia (1)	Relapse (1), CR (2) after a median follow-up of 25 m
Tadic et al. (2017)[37]	1	71	M	NA	Right atrium, tricuspid	Heart failure	NA	Endomyocardial biopsy	Chemotherapy	R-CHOP(CR)	NA	Complete atrioventricular block	PFS>24m

Reference, year	No. patients	Age	Sex	Pathological type	Localization in heart	Symptoms	Arrhythmias	Pathological Diagnostic tool	Therapy	First-line Chemotherapy (Response)	Salvage Chemotherapy	Cardiac complications	Outcome
					valve, right ventricle								
Hu et al. (2017)[38]	1	73	M	Non-GCB	Right atrium	Dyspnea, Chest distress	Third-degree atrioventricular block	Open chest biopsy	Chemotherapy	CHOP（PD）	NA	NA	OS>6m
Hu et al. (2017))[38]	1	27	,F	Non-GCCB	Right atrium, pericardial effusion	Palpitation, chest distress	Atrial fibrillation	Open chest biopsy	Chemotherapy	CHOP（CR）	NA	NA	PFS>20m
Cheng et al. (2018) [39]	1	58	M	GCB	Right atrium, Left ventricle, pericardial effusion	Acute heart failure, Cardiogenic shock, Sudden cardiac death, Hemophagocytosis	Atrial fibrillation, ventricular fibrillation	Endomyocardial biopsy(negative), Open chest biopsy	Chemotherapy	R-CHOP	NA	Bacteremia and sepsis	Died after 2 weeks
Rooijen et al. (2017)[40]	1	69	M	GCB	Right atrium and right ventricle	Superior vena cava syndrome	Ventricular tachycardia, atrial fibrillation	CT-guided biopsy	Chemotherapy	R-CEOP(PR)	NA	CNS relapse	PFS=10m OS=16m
Rooijen et al. (2017)[40]	1	73	M	NA	Right atrium and right ventricle	Syncope	Atrial fibrillation, first degree AV block	Intracardiac echocardiography guided biopsy	Chemotherapy	R-CHOP(CR)	NA	CNS relapse	PFS=24m, OS=26m
Coulier et al (2018) [41]	1	78	F	NA	Interventricular septum, Epicardial effusion	Chest pain	Complete atrioventricular block	Endomyocardial biopsy	Chemotherapy	R-CHOP	NA	NA	NA

Reference, year	No. patients	Age	Sex	Pathological type	Localization in heart	Symptoms	Arrhythmias	Pathological Diagnostic tool	Therapy	First-line Chemotherapy (Response)	Salvage Chemotherapy	Cardiac complications	Outcome
Perrone et al (2018) [42]	1	64	F	NA	Right atrium, right ventricle, tricuspid valve	Dyspnea, cough, asthenia	NA	Endomyocardial biopsy	Chemotherapy	NA	NA	NA	NA
Garagoli et al. (2018) [43]	1	74	F	NA	Thickening of the ventricular and atrial walls, pericardial effusion	Dyspnea, weakness, heart failure,	Atrial fibrillation	Pericardial fluid cytology	None	None	NA	NA	OS<1m
Wang et al. (2018)[44]	1	55	M	NA	Left ventricular, pericardial effusion	Palpitations, chest distress	None	Open chest biopsy	Chemotherapy+ Resection	R-CHOP(CR)	NA	NA	PFS>12m
Endo et al. (2018) [45]	1	79	M	NA	Right atrium, right ventricular	Dyspnea, leg edema, B symptoms (Weight loss)	None	Open chest biopsy	Chemotherapy	R-CHOP(CR)	NA	NA	PFS>5m
Ito et al. (2018)[46]	1	79	M	NA	Right atrium, right ventricle	Dyspnea	Complete heart block	Open chest biopsy	Chemotherapy	R-CHOP	NA	NA	OS<1m
Grantomo et al. (2018)[47]	1	73	M	Non-GCB	Right atrium, right ventricle, pericardial effusion	Dyspnea, syncope symptoms (weight loss)	First grade atrioventricular (AV) block	Open chest biopsy	Palliative	NA	NA	NA	OS=1.5m

Reference, year	No. patients	Age	Sex	Pathological type	Localization in heart	Symptoms	Arrhythmias	Pathological Diagnostic tool	Therapy	First-line Chemotherapy (Response)	Salvage Chemotherapy	Cardiac complications	Outcome
Thiagaraj et al.(2018)[48]	1	50	F	NA	Left ventricle	Abdominal pain, B symptoms	NA	Open chest biopsy	Chemotherapy	R-EPOCH(CR)	NA	NA	PFS>12m
Anand et al. (2019)[49]	1	69	M	Non-GCB	Right atrium, right ventricle, and left ventricle	Dyspnea	Atrial fibrillation complete atrioventricular block status	Endomyocardial biopsy	Chemotherapy	R-EPOCH	NA	NA	PFS>26m
Pollak et al. (2019)[50]	1	81	M	NA	Right atrium, right ventricle	Cough, bilateral leg edema, B symptoms (weight loss), dyspnea		An imaging-guided anterior thoracic node biopsy	Chemotherapy+ radiotherapy	R-CEPO(PR)	NA	NA	OS>1y
Bonou et al. (2019)[51]	1	28	M	GCB	Both atrium, Interatrial septum, Pericardium, pericardial effusion	Dyspnea, Superior vena cava syndrome	No	Endomyocardial biopsy	Chemotherapy	R-CHOP(CR)	NA	Arrhythmias with pauses of up to 12 s	PFS>15m
Natalia et al. (2019)[52]	1	64	F	Non-GCB	Right atrium, right ventricle, tricuspid Valve	Dyspnea, heart failure	Atrial fibrillation	Open chest biopsy	Chemotherapy	R-CHOP(PD)	DHAP	NA	PFS=3m OS>60m
Leef et al. (2020)[53]	1	47	M	NA	Biventricular thickening	Chest pain, dizziness, dyspnea, cardiogenic	Ventricular tachycardia	Endomyocardial biopsy	Chemotherapy	COP(PD)	MTX+ R-EPOCH(CR)	CNS relapse	PFS:1m OS>12m

Reference, year	No. patients	Age	Sex	Pathological type	Localization in heart	Symptoms	Arrhythmias	Pathological Diagnostic tool	Therapy	First-line Chemotherapy (Response)	Salvage Chemotherapy	Cardiac complications	Outcome
						shock							
Meyer et al. (2020)[54]	1	59	F	NA	Interatrial septum, right ventricle	Dyspnea, palpitations	Third-degree atrioventricular block	Open chest biopsy	Chemotherapy	R-CHOP(CR)	NA	NA	PFS>2m
Wang et al. (2020)[55]	1	58	M	NA	Right atrium, pericardial effusion	Chest pain, Dyspnea	Complete heart block	Pericardial fluid cytology	Chemotherapy	R-EPCOH	NA	NA	NA
Jogani et al, (2020) [56]	1	60	M	NA	Right ventricle, right atrium	Heart failure, pulmonary embolism	NA	Echo-guided right heart catheter biopsy	Chemotherapy	R-CHOP(PR)	NA	NA	PFS>3m
Yuce et al. (2020) [57]	1	63	F	NA	Right atrium, pericardial effusion	Dyspnea, weight loss, leg edema, fatigue	None	Endomyocardial biopsy(Negative, CT-guided percutaneous transthoracic biopsy)	Chemotherapy	NA	NA	NA	NA
Azzu et al. (2020)[58]	1	58	F	Non-GCB	Right atrium, right ventricular, pericardial effusion	Dyspnea, superior vena cava obstruction	NA	Endomyocardial biopsy	Chemotherapy	R-CHOP(CR)	NA	NA	PFS>6m
Bhagani et al.(2021)[59]	1	70	F	NA	Right atrium, Right ventricle	Dyspnea	Atrial fibrillation	Imaging guided biopsy	Chemotherapy	R-CHOP(CR)	NA	NA	PFS>5m

Reference, year	No. patients	Age	Sex	Pathological type	Localization in heart	Symptoms	Arrhythmias	Pathological Diagnostic tool	Therapy	First-line Chemotherapy (Response)	Salvage Chemotherapy	Cardiac complications	Outcome
Xia et al. (2021) [60]	1	66	F		Pericardial effusion, Right atrium, Right ventricle	Cardiac tamponade	None	Open chest biopsy	Chemotherapy	NA	NA	NA	PFS>12m
Kane et al. (2022)[61]	1	61	M	NA	Right ventricular, intraventricular septum	Cardiac arrest	NA	Endomyocardial biopsy	Chemotherapy	R-CVP/R-CHOP(CR)	NA	NA	PFS>24m
Li et al. (2022) [62]	1	64	M	Non-GCB	Right atrium, right ventricle, pericardial and pleural effusion	Dyspnea, chest pain,	Atrial fibrillation	NA	NA	NA	NA	NA	OS=3 days
Zhang et al. (2022) [63]	1	66	M	NA	Right atrium, left atrium, left ventricular, interatrial septum, pericardial effusion	Dyspnea, Chest tightness, Cough	NA	Ultrasound-guided percutaneous core needle Biopsy	Chemotherapy	NA	NA	NA	PFS>6m
Bodor et al. (2022)[64]	1	85	M	NA	Pericardial effusion, right ventricular	Dyspnea, Cough, wheeze, heart failure	Transient third-degree heart block	Ultrasound-guided percutaneous biopsy	Chemotherapy	R-CP(PD)	NA	NA	OS=3m
Kim et al. (2020)[65]	1	43	M	NA	Right atrium	Palpitations, dizziness	Second degree atrioventricular block, ventricular tachycardia	Mediastinal lymph node excision biopsy	Chemotherapy	R-CHOP	NA	NA	PFS>9m
Yang et al.	1	79	F	NA	Right	Chest pain,	None	Pericardial	NA	NA	NA	NA	OS=12m

Reference, year	No. patients	Age	Sex	Pathological type	Localization in heart	Symptoms	Arrhythmias	Pathological Diagnostic tool	Therapy	First-line Chemotherapy (Response)	Salvage Chemotherapy	Cardiac complications	Outcome
(2022)[66]					atrium, pericardial effusion	fever		fluid cytology					
Hu et al. (2022) [67]	1	70	M	NA	Left atrium, right atrium, pericardial effusion	Dyspnea, palpitation	Complete AVB	CT-guided percutaneous biopsy	Chemotherapy	R-DAEPOCH(PR)	NA	NA	PFS>6m
Perriello et al. (2023)[68]	1	63	M	Non-GCB	Right atrium, Right ventricle	Syncope	Supraventricular tachycardia	A cardiac biopsy	Chemotherapy	R-CODP(PR)	R-DHAP(PD); Pola-BR, CAR-T	NA	PFS=4m
Kassab et al. (2023)[69]	1	64	M	Non-GCB	Right atrium	Dyspnea, superior vena cava syndrome	NA	Open chest biopsy	Chemotherapy	R-CHOP	NA	NA	NA
Vogl et al. (2023) [70]	1	75	M	NA	Right ventricle	Palpitation, Weakness, chest pain, dyspnea	NA	Endomyocardial biopsy (Negative), CT-guided puncture	Chemotherapy	NA	NA	NA	PFS>10m
Imataki et al. (2023)[71]	1	62	M	NA	Right atrium,	Dyspnea, cardiac failure	Fibrillation rhythm	Open chest biopsy	Chemotherapy	CHOP(CR)	NA	Na	PFS>5m
Bekki et al. (2024)[72]	1	70	F	NA	Right atrium, tricuspid valve	Fatigue, dyspnea	No	Epicardial punch biopsy	Chemotherapy	RCHOP+REPOCH(CR)	NA	NA	NA
Dong et al (2024) [73]	1	60	M	Non-GCB	Left atrium	Chest distress	NA	Open chest biopsy	Resection	Resection	R-CHOP	CNS relapse	PFS=36m OS>114m
Liu et al (2024)[74]	1	64	M	Non-GCB	Right atrium, right ventricle,	Dyspnea	Third-degree atrioventricular block	Open chest biopsy	Chemotherapy	R-CHOP（PR）	NA	NA	OS=3m

Reference, year	No. patients	Age	Sex	Pathological type	Localization in heart	Symptoms	Arrhythmias	Pathological Diagnostic tool	Therapy	First-line Chemotherapy (Response)	Salvage Chemotherapy	Cardiac complications	Outcome
					tricuspid valve, pericardial effusion,								
Rodrigo et al. (2024) [75]	1	69	M	GCB	Right ventricle	Dyspnea, chest pain	NA	Endomyocardial biopsy	Chemotherapy	NA	NA	NA	NA
Kelleher et al. (2024) [76]	1	89	F	Non-GCB	Right atrium, pericardial effusion	Chest pain, dyspnea, Cardiac tamponade	Complete heart block	Endomyocardial biopsy	NA	NA	NA	NA	OS=1m

Notes: NA: not available; F: Female; M: Male; PFS: Progression free survival; OS: Overall survival; GCB: Germinal center B-cell-like lymphoma; non-GCB: non-germinal center B-cell-like lymphoma

References

1. Su, H.-Y., et al., *Primary cardiac lymphoma evaluated with integration of PET/CT and contrast-enhanced CT.* Clinical Nuclear Medicine, 2009. **34**(5): p. 298-301.
2. Dellas, C., et al., *A rare cause of sudden cardiac arrest: primary cardiac lymphoma.* Clinical Research In Cardiology : Official Journal of the German Cardiac Society, 2009. **98**(8): p. 509-511.
3. Utsunomiya, D., et al., *Primary cardiac lymphoma: computed tomography and magnetic resonance imaging features.* Japanese Journal of Radiology, 2009. **27**(6): p. 243-246.
4. Sankaranarayanan, R. and K. Prasanna, *A case of primary cardiac lymphoma mimicking acute myocardial infarction.* Clinical Cardiology, 2009. **32**(8): p. E52-E54.
5. Schell, A.J., et al., *Primary cardiac lymphoma: molecular cytogenetic characterization of a rare entity.* Cardiovascular Pathology : the Official Journal of the Society For Cardiovascular Pathology, 2009. **18**(2): p. 92-99.
6. Patel, J., L. Melly, and M.N. Sheppard, *Primary cardiac lymphoma: B- and T-cell cases at a specialist UK centre.* Annals of Oncology : Official Journal of the European Society For Medical Oncology, 2010. **21**(5): p. 1041-1045.
7. Mariani, J.A., et al., *Primary cardiac lymphoma.* Journal of the American College of Cardiology, 2010. **55**(13): p. e23.

8. Park, S.M., et al., *Coronary sinus obstruction by primary cardiac lymphoma as a cause of dyspnea due to significant diastolic dysfunction and elevated filling pressures.* Journal of the American Society of Echocardiography : Official Publication of the American Society of Echocardiography, 2010. **23**(6): p. 682.e5-682.e7.

9. Cho, J.-M., I.-S. Sohn, and Y.-J. Yang, *Heart in the heart: dual faced primary cardiac lymphoma on PET-CT.* International Journal of Cardiology, 2010. **142**(3): p. e40-e41.

10. Cho, S.-F., et al., *Primary cardiac lymphoma mimicking atrial thrombus in a patient who underwent permanent pacemaker implantation.* Annals of Hematology, 2011. **90**(6): p. 739-740.

11. Cho, S.W., et al., *Primary cardiac lymphoma presenting with atrioventricular block.* Korean Circulation Journal, 2010. **40**(2): p. 94-98.

12. Lee, J.C., et al., *Positron emission tomography combined with computed tomography as an integral component in evaluation of primary cardiac lymphoma.* Clinical Cardiology, 2010. **33**(6): p. E106-E108.

13. Chen, X., et al., *Primary cardiac lymphoma complicated with inferior vena cava thrombosis: a case report.* Acta Cardiologica, 2010. **65**(3): p. 347-349.

14. Nishizawa, M., et al., *Neurolymphomatosis as a manifestation of relapsed primary cardiac lymphoma.* International Journal of Hematology, 2010. **92**(5): p. 679-680.

15. Lin, J.N., *Cardiac lymphoma with first manifestation of recurrent syncope-a case report and literature review.* International Medical Case Reports Journal, 2010. **3**: p. 1-6.

16. Minamimoto, R., et al., *Value of FDG-PET/CT using unfractionated heparin for managing primary cardiac lymphoma and several key findings.* Journal of Nuclear Cardiology : Official Publication of the American Society of Nuclear Cardiology, 2011. **18**(3): p. 516-520.

17. Bambury, R., et al., *Primary cardiac lymphoma: diagnostic tools and treatment challenges.* Irish Journal of Medical Science, 2011. **180**(1): p. 271-273.

18. Matsushita, T., et al., *Cardiac xanthoma originating from primary cardiac lymphoma.* The Annals of Thoracic Surgery, 2012. **94**(6): p. 2120-2122.

19. Yoshihara, S., et al., *A case of primary cardiac lymphoma: in vivo imaging and pathologic correlation.* European Heart Journal. Cardiovascular Imaging, 2013. **14**(10): p. 1027.

20. Oka, T., et al., *Primary cardiac lymphoma diagnosed by endomyocardial biopsy using transthoracic echocardiography in the substernal window.* Journal of Medical Ultrasonics (2001), 2013. **40**(4): p. 483-485.

21. Kawamura, T., et al., *Successful treatment of a large primary cardiac lymphoma by surgical resection combined with chemotherapy: report of a case.* Surgery

Today, 2013. **43**(9): p. 1066-1070.

22. Shah, K. and K. Shemisa, *A "low and slow" approach to successful medical treatment of primary cardiac lymphoma.* Cardiovascular Diagnosis and Therapy, 2014. **4**(3): p. 270-273.

23. Kim, D.-H., et al., *Primary cardiac lymphoma presenting as an atypical type of hypertrophic cardiomyopathy.* Echocardiography (Mount Kisco, N.Y.), 2014. **31**(4): p. E115-E119.

24. Jung, Y.H., et al., *A case of primary cardiac lymphoma showing isolated central nervous system relapse.* Clinical Lymphoma, Myeloma & Leukemia, 2014. **14**(1): p. e31-e33.

25. Montoro, J., et al., *Primary cardiac lymphoma with isolated parenchymal central nervous system relapse: report of two cases and review of the literature.* Ecancermedicalscience, 2014. **8**: p. 474.

26. Nijjar, P.S., et al., *Benefits and limitations of multimodality imaging in the diagnosis of a primary cardiac lymphoma.* Texas Heart Institute Journal, 2014. **41**(6): p. 657-659.

27. Jonavicius, K., et al., *Primary cardiac lymphoma: two cases and a review of literature.* Journal of Cardiothoracic Surgery, 2015. **10**: p. 138.

28. Riccioli, V., et al., *Collateral Circulation Resulting From Obstruction Due to Cardiac Lymphoma of Right Atrium.* Clinical Lymphoma, Myeloma & Leukemia, 2015. **15**(11): p. e173-e176.

29. Jandali, A., et al., *Rare cause of a common symptom: primary cardiac lymphoma.* The American Journal of Emergency Medicine, 2015. **33**(12): p. 1849.e5-1849.e6.

30. Hong, T.H. and D.S. Jeong, *Successful Management of Primary Cardiac Lymphoma with Minimal Debulking Surgery Combined with Adjuvant Chemotherapy.* The Heart Surgery Forum, 2015. **18**(6): p. E242-E244.

31. Pagé, M., et al., *Primary Cardiac Lymphoma: Diagnosis and the Impact of Chemotherapy on Cardiac Structure and Function.* The Canadian Journal of Cardiology, 2016. **32**(7): p. 931.e1-931.e3.

32. Moss, E., et al., *Successful Robotic Excision and Early Chemotherapy for Primary Cardiac Lymphoma.* The Annals of Thoracic Surgery, 2016. **102**(1): p. 304-305.

33. Liu, Y., et al., *Primary cardiac lymphoma: Two rare cases.* International Journal of Cardiology, 2016. **203**: p. 763-765.

34. Ciancarella, P., et al., *Multimodality imaging evaluation of a primary cardiac lymphoma.* Journal of the Saudi Heart Association, 2017. **29**(2): p. 128-135.

35. Singh, B., et al., *Primary Cardiac Lymphoma: Lessons Learned from a Long Survivor.* Case Reports In Cardiology, 2016. **2016**: p. 7164829.

36. Cereda, A.F., et al., *Impact of serial echocardiography in the management of primary cardiac lymphoma.* Journal of the Saudi Heart Association, 2018. **30**(2): p. 160-163.

37. Tadic, M., et al., *Cardiac Lymphoma: After the Hurricane.* Chinese Medical Journal, 2017. **130**(19): p. 2391-2392.

38. Hu, S., et al., *Imaging Features of Primary Cardiac Lymphoma.* Chinese Medical Journal, 2017. **130**(17): p. 2123-2125.

39. Cheng, J.-F., et al., *Fulminant primary cardiac lymphoma with sudden cardiac death: A case report and brief review.* Journal of the Formosan Medical Association = Taiwan Yi Zhi, 2018. **117**(10): p. 939-943.

40. van Rooijen, C.R., et al., *Primary cardiac lymphoma with central nervous system relapse.* Clinical Case Reports, 2017. **5**(9): p. 1454-1458.

41. Coulier, B., et al., *Imaging features of primary cardiac lymphoma.* Diagnostic and Interventional Imaging, 2018. **99**(2): p. 115-117.

42. Perrone, M.A., et al., *Primary cardiac lymphoma: the role of multimodality imaging.* Journal of Cardiovascular Medicine (Hagerstown, Md.), 2018. **19**(8): p. 455-458.

43. Garagoli, F., et al., *Cardiac Lymphoma: A Rare Cause of Acute Heart Failure with Restrictive Physiology.* Arquivos Brasileiros de Cardiologia, 2018. **110**(2): p. 203-204.

44. Wang, S., et al., *Multimodal imaging evaluation of a primary cardiac lymphoma in an immunocompetent patient.* Echocardiography (Mount Kisco, N.Y.), 2018. **35**(12): p. 2121-2123.

45. Endo, Y., et al., *Treatment of malignant primary cardiac lymphoma with tumor resection using minimally invasive cardiac surgery.* Journal of Cardiothoracic Surgery, 2018. **13**(1): p. 97.

46. Ito, I., et al., *Primary Cardiac Lymphoma: A Lesson Learned from an Unsuccessful Experience.* Internal Medicine (Tokyo, Japan), 2018. **57**(24): p. 3569-3574.

47. Grantomo, J., et al., *A rare case of primary cardiac lymphoma and the role of early surgical debulking: a case report.* European Heart Journal. Case Reports, 2018. **2**(4): p. yty116.

48. Thiagaraj, A., et al., *An unprecedented case report of primary cardiac lymphoma exclusive to left ventricle: a diagnostic and therapeutic challenge.* European Heart Journal. Case Reports, 2018. **2**(2): p. yty029.

49. Anand, K., et al., *Complete Response to R-EPOCH in Primary Cardiac Lymphoma.* Case Reports In Hematology, 2019. **2019**: p. 7690430.

50. Pollak, P.T., et al., *Treatment Decisions in Geriatric Cardiac Lymphoma Facilitated by Serial Cardiac Magnetic Resonance Imaging and Positron Emission*

Tomography. CJC Open, 2019. **1**(3): p. 153-157.

51. Bonou, M., et al., *Diagnosis and treatment complications of primary cardiac lymphoma in an immunocompetent 28-year old man: a case report.* BMC Cancer, 2019. **19**(1): p. 191.
52. Cichowska-Cwalińska, N., et al., *The role of radiotherapy in the management of primary cardiac lymphoma a case report and the literature review.* Leukemia & Lymphoma, 2019. **60**(3): p. 812-816.
53. Leef, G., D.E. Gladstone, and O. Cingolani, *ECMO Therapy for Cardiac Lymphoma.* Circulation, 2020. **142**(12): p. 1219-1223.
54. Meyer, M., et al., *Primary cardiac lymphoma.* European Heart Journal. Cardiovascular Imaging, 2020. **21**(7): p. 816.
55. Wang, S.C. and C.W. Pan, *Overcoming the diagnostic challenges in a high-risk invasive primary cardiac lymphoma.* European Heart Journal. Cardiovascular Imaging, 2020. **21**(6): p. 707.
56. Jogani, S., et al., *Pulmonary embolism and primary cardiac lymphoma.* Acta Cardiologica, 2020. **75**(7): p. 689-690.
57. Yuce, G. and A. Coskun, *An unusual case of cardiac lymphoma diagnosed using computed tomographyguided percutaneous transthoracic biopsy.* Anatolian Journal of Cardiology, 2020. **24**(1): p. 59-61.
58. Azzu, A., et al., *A case report of a primary cardiac lymphoma causing superior vena cava obstruction: the value of multimodality imaging in the clinical workup.* European Heart Journal. Case Reports, 2020. **4**(6): p. 1-5.
59. Bhagani, S., et al., *Complete regression of diffuse large B-cell cardiac lymphoma: the role of multimodality cardiovascular imaging in diagnosis and assessing treatment response.* European Heart Journal. Cardiovascular Imaging, 2021. **22**(4): p. e10.
60. Xia, J., et al., *Surgical treatment of a primary cardiac lymphoma presenting with cardiac tamponade.* General Thoracic and Cardiovascular Surgery, 2021. **69**(2): p. 356-359.
61. Kane, C., et al., *Incidental finding of primary cardiac lymphoma after cardiac arrest and percutaneous coronary intervention.* European Heart Journal. Cardiovascular Imaging, 2022. **23**(9): p. e327.
62. Li, J., et al., *Diagnosis of rapidly progressed primary cardiac lymphoma in liver transplant recipient: A case report.* Frontiers In Oncology, 2022. **12**: p. 1014371.
63. Zhang, D.-L., et al., *Primary Cardiac Lymphoma Diagnosed by Ultrasound-Guided Percutaneous Core Needle Biopsy.* Journal of Vascular and Interventional Radiology : JVIR, 2022. **33**(5): p. 611-614.

64. Bodor, T.L.W., J.C. Webber, and J.A. Scheske, *Primary Cardiac Lymphoma in an 85-Year-Old Man, With Highly Suggestive Features on Imaging.* CJC Open, 2023. **5**(2): p. 167-169.

65. Kim, S., et al., *A Long Journey to the Truth: Primary Cardiac Lymphoma with Various Arrhythmias from Ventricular Tachycardia to Atrial Flutter.* Korean Circulation Journal, 2020. **50**(4): p. 374-378.

66. Yang, D., et al., *Cardiac Lymphoma Diagnosed by Multi-Modality Imaging: A Case Report.* Frontiers In Cardiovascular Medicine, 2022. **9**: p. 771538.

67. Hu, B., et al., *A case of complete atrioventricular block associated with primary cardiac lymphoma reversed without cardiac pacemaker implantation.* The Journal of International Medical Research, 2022. **50**(4): p. 3000605221089780.

68. Perriello, V.M., et al., *CAR T cell-driven cerebrospinal fluid cytokine storm with confounding neurological picture in chemorefractory primary cardiac lymphoma.* American Journal of Hematology, 2023. **98**(1): p. 212-219.

69. Kassab, J., et al., *Case report: Primary cardiac lymphoma manifesting as superior vena cava syndrome.* Frontiers In Cardiovascular Medicine, 2023. **10**: p. 1257734.

70. Vogl, T.J., et al., *Letter to the Editor: CT Guided Biopsy of a Right Ventricle Primary Cardiac Lymphoma-A Case Report.* Cardiovascular and Interventional Radiology, 2023. **46**(7): p. 970-972.

71. Imataki, O., et al., *Metabolic Steal of the Myocardium by Primary Cardiac Lymphoma.* Case Reports In Oncology, 2023. **16**(1).

72. Bekki, M., N. Tahara, and Y. Fukumoto, *Primary cardiac lymphoma causing coronary artery compression and massive tricuspid regurgitation.* European Heart Journal, 2024. **45**(34): p. 3185.

73. Dong, W. and W. Li, *Brain metastases from primary cardiac lymphoma: A case report.* Asian Journal of Surgery, 2024.

74. Liu, J., et al., *Case Report: A case of third-degree atrioventricular block associated with primary cardiac lymphoma.* Frontiers In Cardiovascular Medicine, 2024. **11**: p. 1356134.

75. Ugueto Rodrigo, C., L. Fernández Gassó, and S. Jiménez Valero, *Cardiac lymphoma as a cause of dyspnea in an immunosuppressed patient: the importance of the endomyocardial biopsy.* The Journal of Invasive Cardiology, 2024. **36**(8).

76. Kelleher, R., et al., *Primary cardiac lymphoma presenting with cardiac tamponade and complete heart block: case report.* European Heart Journal. Case Reports, 2024. **8**(1): p. ytad635.
